# High Resolution Proteomic Analysis of Subcellular Fractionated Boar Spermatozoa Provides Comprehensive Insights Into Perinuclear Theca-Residing Proteins

**DOI:** 10.3389/fcell.2022.836208

**Published:** 2022-02-18

**Authors:** Min Zhang, Riccardo Zenezini Chiozzi, David A. Skerrett-Byrne, Tineke Veenendaal, Judith Klumperman, Albert J. R. Heck, Brett Nixon, J. Bernd Helms, Bart M. Gadella, Elizabeth G. Bromfield

**Affiliations:** ^1^ Department of Biomolecular Health Sciences and Department of Farm and Animal Health, Faculty of Veterinary Medicine, Utrecht University, Utrecht, Netherlands; ^2^ Biomolecular Mass Spectrometry and Proteomics, Bijvoet Centre for Biomolecular Research and Utrecht Institute for Pharmaceutical Sciences, Utrecht University, Utrecht, Netherlands; ^3^ Netherlands Proteomics Centre, Utrecht, Netherlands; ^4^ Structural Biochemistry, Bijvoet Centre for Biomolecular Research, Utrecht University, Utrecht, Netherlands; ^5^ Priority Research Centre for Reproductive Science, School of Environmental and Life Sciences, Discipline of Biological Sciences, University of Newcastle, Callaghan, NSW, Australia; ^6^ Section Cell Biology, Center for Molecular Medicine, University Medical Center Utrecht, Utrecht University, Utrecht, Netherlands

**Keywords:** proteomics, spermatozoa, perinuclear theca, sperm function, fertilization

## Abstract

The perinuclear theca (PT) is a highly condensed, largely insoluble protein structure that surrounds the nucleus of eutherian spermatozoa. Recent reports have indicated that the PT unexpectedly houses several somatic proteins, such as core histones, which may be important post-fertilization during re-modelling of the male pronucleus, yet little is known regarding the overall proteomic composition of the PT. Here, we report the first in depth, label-free proteomic characterization of the PT of boar spermatozoa following the implementation of a long-established subcellular fractionation protocol designed to increase the detection of low abundance proteins. A total of 1,802 proteins were identified, a result that represents unparalleled depth of coverage for the boar sperm proteome and exceeds the entire annotated proteome of the *Sus scrofa* species so far. In the PT structure itself, we identified 813 proteins and confirmed the presence of previously characterized PT proteins including the core histones H2A, H2B, H3 and H4, as well as Ras-related protein Rab-2A (RAB2A) and Rab-2B (RAB2B) amongst other RAB proteins. In addition to these previously characterized PT proteins, our data revealed that the PT is replete in proteins critical for sperm-egg fusion and egg activation, including: Izumo family members 1–4 (IZUMO1-4) and phosphoinositide specific phospholipase ζ (PLCZ1). Through Ingenuity Pathway Analysis, we found surprising enrichment of endoplasmic reticulum (ER) proteins and the ER-stress response in the PT. This is particularly intriguing as it is currently held that the ER structure is lost during testicular sperm maturation. Using the String and Cytoscape tools to visualize protein-protein interactions revealed an intricate network of PT protein complexes, including numerous proteasome subunits. Collectively, these data suggest that the PT may be a unique site of cellular homeostasis that houses an abundance of protein degradation machinery. This fits with previous observations that the PT structure dissociates first within the oocyte post-fertilization. It remains to be explored whether proteasome subunits within the PT actively assist in the protein degradation of paternal cell structures post-fertilization and how aberrations in PT protein content may delay embryonic development.

## Introduction

The perinuclear theca (PT) is a unique, highly condensed, cytoskeletal structure that surrounds the nucleus of mature mammalian sperm cells. This structure is non-ionic detergent-resistant and, in most eutherian species, consists of three sub-domains termed the sub-acrosomal layer (SAL), the equatorial segment (ES) and post-acrosomal sheath (PAS) (Oko and Sutovsky, 2009). Originally the PT was thought to provide structural support to the nucleus during the long transit of sperm cells through the male and female reproductive tracts. More recently, the observations that the PAS-PT of the sperm cell is the first internal structure to become exposed in the ooplasm during fertilization has resulted in the PT receiving considerable attention for its potential roles in sperm-egg interaction (Sutovsky et al., 2003; Hamilton et al., 2018) and early events post-fertilization (Wu et al., 2007; Aarabi et al., 2014; Hamilton et al., 2019). Crucially, in some species the PAS-PT has been proposed to harbor the sperm-oocyte activation factor(s) that are released post-fertilization in the ooplasm and are then responsible, at least in part, for triggering Ca^2+^ oscillations within the oocyte (Sutovsky et al., 2003; Hamilton et al., 2018).

While the identity of these egg activation factors has been long contested, current evidence appears to rest with the activity of two phosphoinositide specific phospholipase ζ (PLCZ1) (Cox et al., 2002) and post-acrosomal WW domain-binding protein (PAWP, also known as WBP2NL) (Wu et al., 2007; Nakai et al., 2020). Both PLCZ1 and PAWP have been found to reside within the PT in some species, generating the hypothesis that the PT may be enriched in proteins important for fertilization (Oko and Sutovsky, 2009; Zafar et al., 2021). Moreover, experiments conducted to determine the precise region of the sperm cell capable of activating the oocyte have revealed that when all membranes are removed using Triton X-100, leaving the PT, the sperm cell still retains the capacity for oocyte activation (Kimura et al., 1998). Finally, in the context of male infertility, patients with globozoospermia with an absence of the PAS-PT were infertile due to an inability to activate the oocyte (Ito et al., 2009). This provides critical evidence for the importance of PT-resident proteins in ensuring successful fertilization.

Despite knowledge that the PT is essential for fertilization and that the proteins residing within this structure likely contribute to oocyte activation, a comprehensive proteomic investigation of the PT to understand its full contribution to these processes has yet to be performed. This is likely due to previous limitations in isolating PT proteins and the relatively slow uptake of proteomic technologies to investigate reproductive cells. However, even prior to the widespread use of mass spectrometry to characterize sperm proteins, pioneering work by Oko and Sutovsky and their respective colleagues revealed that the PT houses an unexpected array of proteins including somatic (core) histones (Tovich and Oko, 2003) that appear to be de novo-synthesized during the round spermatid stage of development and are then assembled during spermatid elongation (Hamilton et al., 2021). Remarkably, these histones then appear to play an important role post-fertilization as intra-cytoplasmic sperm injection (ICSI) of sperm cells depleted of PT-histones resulted in a delay in embryonic development (Hamilton et al., 2021). Similarly, the PT-residing protein glutathione S-transferase omega 2 (GSTO2) has recently been shown to accelerate nuclear decondensation of sperm cells during fertilization (Hamilton et al., 2019), implying that multiple PT-enriched proteins may contribute to functions in the zygote.

Given the importance of the PT for fertilization, and our own recent characterization of the PT-residing protein cysteine rich secretory protein 2 (CRISP2) and its role in protein scaffolding in the sperm cell (Zhang et al., 2021), we elected to conduct a comprehensive proteomic analysis of the isolated PT using a quantitative, label-free liquid chromatography mass spectrometry (LC-MS/MS) approach. This approach was able to be applied to PT proteins through the development of highly reproducible methods by the Sutovsky and Oko groups for isolating the PT and its proteins from the sperm head that have been published and used extensively (Aul and Oko, 2002; Tovich and Oko, 2003; Wu et al., 2007; Hamilton et al., 2017). The purpose of this mass spectrometry based-exploration of the PT was to provide a full inventory of PT-residing (proteins present in the PT but also present in other cell compartments) and PT-enriched (proteins with significantly higher abundance in the PT than other compartments) proteins in sperm cells that can be used as a resource for the sperm-egg activation and fusion fields to help shed light on the role of PT proteins during and post-fertilization. Moreover, given the extensive protein crosslinking by intra and inter protein disulfide bridge formation that takes place within the PT structure to ensure its condensed nature prior to egg interaction, we sought to construct a network of PT-enriched proteins that can be further probed to understand protein-protein interactions that take place within the PT. This work was conducted in boar spermatozoa due to the well-characterized nature of the PT in this species, our own work related to CRISP2 in the boar and due to the genetic and physiological relevance of pig reproductive processes to those of humans (Kuzmuk and Schook, 2011; Zuidema and Sutovsky, 2020).

## Experimental Procedures

### Preparation of Boar Spermatozoa

Semen was collected from three mature and highly fertile boars. The collected semen was diluted to 20 million sperm/ml in a commercial diluent, packed in insemination tubes of 80 ml and transported at 17°C to Utrecht University by a courier from a commercial breeder (Cooperative Center for Artificial Insemination in Pigs, Veghel, the Netherlands). Semen samples were used within 12 h. Sperm cells were washed through discontinuous Percoll (GE Healthcare, Piscataway, NJ, United States) gradients as previously described (Zhang et al., 2021).

### Subcellular Fractionation of Boar Sperm Into Head, Tail and Perinuclear Theca Components

**FIGURE 1:**
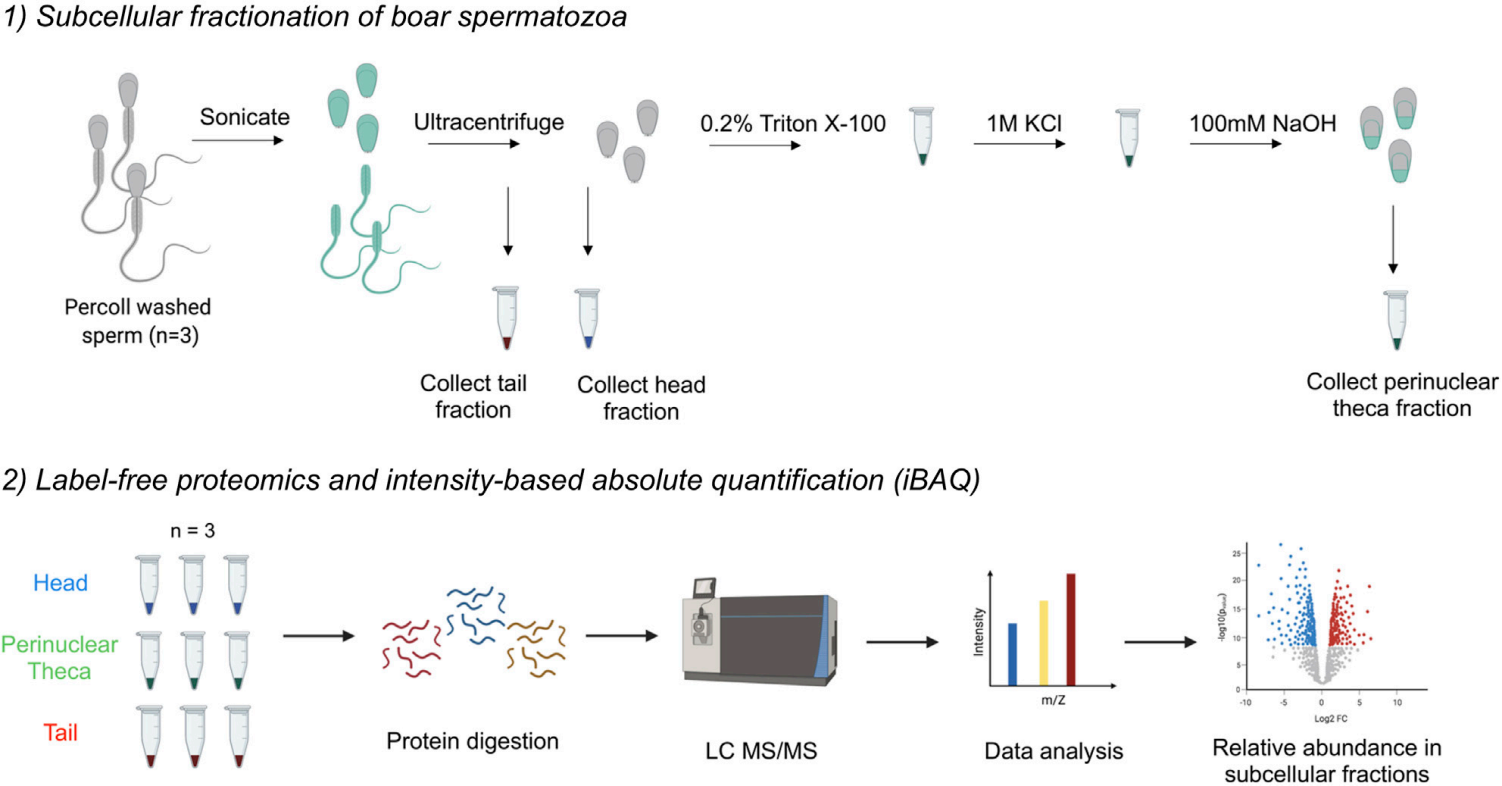
Workflow diagram for the isolation of the perinuclear theca (PT) and application of mass spectrometry-based proteomics to boar spermatozoa. *1*) The separation of sperm heads and tails was performed first through sonication and second, through ultracentrifugation on a 62% sucrose gradient. To isolate the PT structures, boar sperm heads were then subjected to successive extraction steps using 0.2% Triton X100 and 1 M KCl for 1 h each with agitation. The resulting pellets were washed and then incubated in 0.1 M NaOH overnight. The 0.2% Triton X100 solubilizes proteins associated with the inner acrosomal membrane, 1 M KCl releases ionically attached PT proteins and the final 0.1 M NaOH extracts covalently bound PT proteins in accordance with the protocol by Mountjoy et al., 2008). *2*) Once three biological replicates of head, tail and PT material were collected, cell samples were lysed and proteins were digested. Peptides were injected onto an ultra-high-performance liquid chromatography (LC) system coupled to an Orbitrap HF mass spectrometer (MS). Data analysis was performed in MaxQuant to calculate intensity-based absolute quantification (iBAQ) values, and further analysis was conducted using the platforms: Perseus, Ingenuity Pathway Analysis (IPA), String and Cytoscape.

To separate the sperm heads and tails, Percoll washed sperm cells (1 × 10^8^) were resuspended in 1.5 ml PBS with 1 mM phenylmethylsulfonyl fluoride (PMSF) and sonicated on ice at nine microns using an MSE Ltd Soniprep 150 sonicator (East Sussex, United Kingdom) at 15-s bursts with 45-s interval for three cycles until >99% of all sperm heads were detached from tails. Sonicated sperm cells were loaded on a 62% (w/v) sucrose gradient to isolate the sperm heads and tails as previously described (Zhang et al., 2021). Isolation of perinuclear theca (PT) proteins was performed *via* methods originally described by Oko and Maravei, 1994 (Oko and Maravei, 1994) and described in full in Zhang et al., 2021 (Zhang et al., 2021) (for schematic *see* Figure 1). Briefly, purified sperm heads underwent continuous extraction steps in 0.2% (v/v) Triton X-100 and 1 M KCl for 1 h at RT with agitation. Following this incubation, the resulting pellets were washed twice before the next extraction and/or fixed in 4% paraformaldehyde (PFA) for immunodetection. The PT proteins were obtained by incubating the resulting pellets in 0.1 M NaOH overnight at 4°C with agitation. The sperm heads, tails and PT fractions were used directly or frozen at −80°C for later analysis.

### Sodium Dodecyl Sulfate–Polyacrylamide Gel Electrophoresis (SDS-PAGE) and Immunoblotting

PT fractions and the resulting pellets were denatured in 4 × SDS sample buffer (200 mM Tris-HCl, pH 6.8, 10% β-mercapto-ethanol, 8% SDS, 0.08% bromophenol blue, 40% glycerol) by boiling for 10 min. Samples were centrifuged at 14,000 × *g* for 2 min, at RT and loaded on to SDS-PAGE gels (5% stacking gel, 12% resolving gel) and were blotted onto 0.2 μm nitrocellulose membranes (GE Healthcare, Piscataway, NJ, United States) at 100 V for 1 h. After blocking for 3 h at RT in 5% (w/v) BSA in PBS with 0.05% (v/v) Tween-20 (PBST), membranes were incubated with primary antibodies (Supplementary Table S1) overnight at 4°C. After three washes in PBST for 15 min, membranes were incubated with horse radish peroxidase (HRP) conjugated secondary antibodies (Supplementary Table S1) for 1 h at RT. After rinsing four times in PBST for 20 min, membranes were developed using a chemiluminescence ECL-detection kit (Supersignal West Pico, Pierce, Rockford IL, United States). The molecular weight of proteins of interest were established using a PageRule Plus pre-stained protein ladder, 10–250 kDa (Thermo Scientific).

### Immunofluorescence Analysis

Whole sperm cells and sperm heads were fixed in 4% PFA for 15 min at RT and permeabilized using 0.5% (v/v) Triton X-100 for 15 min, at RT. For acrosin staining, sperm cells were fixed and permeabilized in −20°C methanol for 5 min, mixed with PBS and deposited on slides. After rinsing with PBS, slides were blocked with 1% (w/v) BSA in PBS for 1 h at RT, incubated overnight at 4°C with primary antibodies. Slides were washed again before incubation for 1 h at RT with secondary antibodies or 20 min with peanut agglutinin lectin (PNA) (Supplementary Table S1) and a Hoechst 33342 counterstain added for 10 min, at RT (1 μg/ml; Sigma). After extensive washing with PBS, slides were mounted with FluorSave reagent (Merck Millipore) and covered with coverslips. For all negative controls the primary antibody was omitted. Images were taken on a Leica SPE-II confocal microscope using a ×63 objective (NA 1.3, HCX PLANAPO oil) and images were analyzed using ImageJ software (bundled with 64-bit Java 1.8.0_172; National Institutes of Health, Bethesda, MD, United States).

### Immunogold Labelling

Percoll washed whole sperm cells were resuspended in PBS, mixed with an equal volume of 4% PFA and fixed for 5 min at RT. The fixative was removed by centrifugation at 750 × *g*, for 5 min at RT. Fresh 4% PFA was added and cells were then fixed overnight. This 4% PFA was then replaced with 1% PFA and sperm cells were kept at 4°C. Further processing of samples for ultrathin cryo-sectioning and immunolabelling was performed according to the protein A-gold method as described previously (Slot and Geuze, 2007). Briefly, fixed cells were washed with 0.05 M glycine in PBS, resuspended and then pelleted in 12% gelatin in PBS at 37°C. The sperm pellet was solidified on ice and cut into small blocks. For cryoprotection, blocks were infiltrated overnight with 2.3 M sucrose at 4°C. The blocks were mounted on aluminum pins and frozen in liquid nitrogen. To pick up the ultrathin cryosections (60 nm), a 1:1 mixture of 2.3 M sucrose and 1.8% methylcellulose was used. RAB2B and PAWP were detected by 10 nm protein A coupled gold particles (Cell Microscopy Core, UMC Utrecht, the Netherlands) and stained with 2% uranyl acetate oxalate and 0.4% uranyl acetate in methylcellulose to increase contrast. EM imaging was performed using a JEOL 1011 microscopy.

### Sample Preparation for Mass Spectrometry

Three biological replicates for sperm heads, tails and PT fractions were lysed following the protocol described by Leung et al., 2021 (Leung et al., 2021) for intact sperm cells. The lysis buffer contained 100 mM Tris-HCl pH 8.5, 7 M Urea, 1% Triton X-100, 5 mM tris-2 (-carboxyethyl)-phosphine (TCEP), 30 mM chloroacetamide (CAA), 10 U/ml DNase I, 1 mM MgCl_2_, 1% benzonase (Merck Millipore, Darmstadt, Germany), 1 mM sodium orthovanadate, phosphoSTOP phosphatases inhibitors and Complete Mini EDTA-free protease inhibitors. Samples were sonicated on ice for 2 min using an ultrasonic processor (UP100H, Hielscher) at 80% amplitude. The proteins were then precipitated with chloroform/methanol and the dried protein pellet resuspended in digestion buffer (100 mM Tris-HCl pH 8.5, 1% sodium deoxycholate (Sigma-Aldrich), 5 mM TCEP, and 30 mM CAA). Trypsin and Lys-C proteases were added to a 1:25 and 1:100 ratio (w/w) respectively and protein digestion performed overnight at 37°C. The final peptide mixtures were desalted with solid-phase extraction C18 columns (Sep-Pak, Waters).

### Liquid Chromatography/Mass Spectrometry

1,000 ng of peptides from each biological replicate were first injected onto an Agilent 1,290 Infinity UHPLC system on a 50-cm analytical column packed with C18 beads (Agilent Poroshell EC-C18, 2.7 μm, 50 cm × 75 μm) coupled online to an Orbitrap HF (Thermo Fisher Scientific). The LC-MS settings were used as in the article of Hidalgo-Gutiérrez et al., 2021 (Hidalgo-Gutiérrez et al., 2021) with minor modifications. After 5 min of loading with 100% buffer A (H_2_O with 0.1% formic acid), peptides were eluted at 300 nl/min with a 95-min gradient from 10 to 40% of buffer B (80% acetonitrile and 20% H_2_O with 0.1% formic acid). For MS acquisition, we used an MS1 Orbitrap scan at 120,000 resolution, automatic gain control (AGC) target of 3 × 10^6^ ions and maximum inject time of 120 ms from 310 to 1,600 m/z; the 15 most intense ions were submitted to MS2 Orbitrap scan at 30,000 resolution, AGC target of 1 × 10^5^ ions and maximum inject time of 54 ms (isolation window of 1.4 m/z, NCE at 28% and dynamic exclusion of 16 s). The proteomic approach undertaken in this manuscript is presented visually in Figure 1.

### Proteomic Data Processing and Analysis

The raw files were analyzed with MaxQuant (version 1.6.17) with all the default settings adding deamidation (NQ) as dynamic modification against the *Sus scrofa* reference proteome (UniProt version of 08/2020 with 49,795 entries) adding common contaminants. MaxQuant was used with the standard parameters adding only the “iBAQ Quantification” and “Match between runs” with automatic values.

The protein quantification generated from MaxQuant without “reverse” and “potential contaminants” were analyzed using Perseus (version 1.6.8.0) (Tyanova et al., 2016). Only proteins with a quantitative value in all three replicates within at least one group, and ≥1 razor plus unique peptides were kept for further analysis. Log transformation and data normalization steps were performed using Perseus. Fold changes and significance calculations between the three sample groups “head,” “tail,” and “perinuclear theca,” were also generated in Perseus for the production of volcano plots, principal component analyses and heat maps. These data were exported from Perseus and plotted using GraphPad Prism (version 8.4.1). Using OmicsBox (version 1.3.11, BioBam Bioinformatics, Valencia, Spain), human homologues for each boar protein were mapped as described previously (Skerrett-Byrne et al., 2021a). For the analysis of pathways enriched in each sample group, Ingenuity Pathway Analysis software (Qiagen) was applied to the refined human homolog proteomic lists, as described in Skerrett-Byrne et al*.*, 2021 (Skerrett-Byrne et al., 2021b; Skerrett-Byrne et al., 2021c). Canonical pathway and disease and function analyses were generated and ranked by Z-score enrichment with a significance cut off of ≥2 for activation and ≤−2 for inhibition. Enriched protein networks were generated using String version 11.5 (Szklarczyk et al., 2021) and modified in Cytoscape (version 3.8.2). These networks were prepared from refined protein lists for each sample group featuring all proteins significantly enriched in each subcellular fraction (*p* value ≤ 0.05; fold change ≥ 2) and those featuring uniquely in each subcellular fraction.

## Results

### Isolation of the Sperm Perinuclear Theca and Assessment of Head and Tail Separation

**FIGURE 2:**
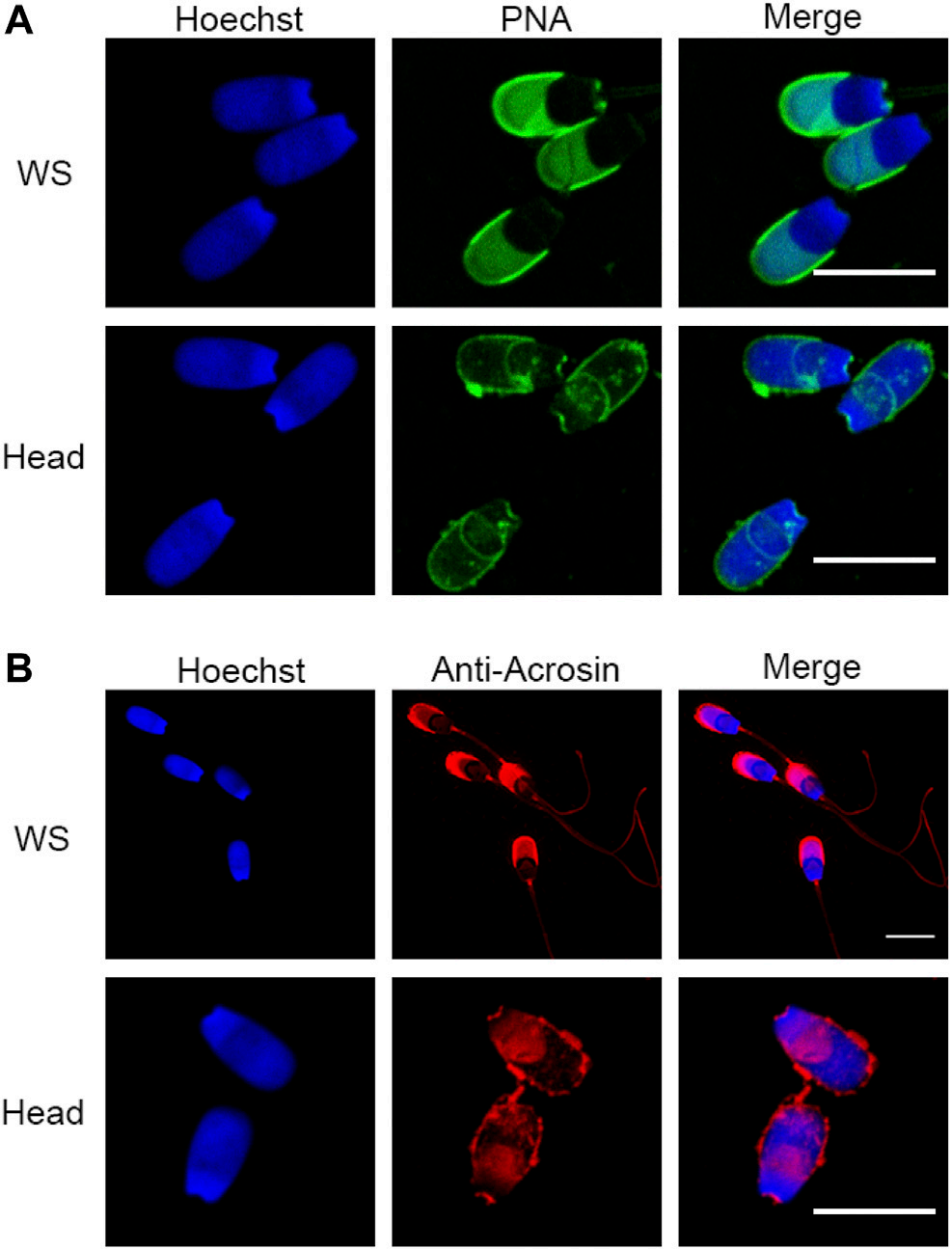
Assessment of sperm head and tail separation using immunofluorescence. **(A)** Immunofluorescence staining with peanut agglutinin lectin (PNA) in whole sperm cells (WS) and fractionated sperm heads. **(B)** Immunofluorescence of acrosin in WS and fractionated sperm heads. Scale bar =  10 μm.

To perform a definitive proteomic characterization of the perinuclear theca structure of the boar spermatozoa, an established method developed by Oko and Maravei was used to isolate the highly dense PT structures from sperm heads (Oko and Maravei, 1994). This method has been used extensively to characterize individual proteins residing within the PT (Korley et al., 1997; Lécuyer et al., 2000; Aul and Oko, 2002; Hamilton et al., 2017; Zhang et al., 2021). To simultaneously provide a proteomic characterization of the sperm head and tail for comparative purposes, the heads and tails of the cells were separated as described previously (Zhang et al., 2021). Although phase-contrast microscopy revealed populations of morphologically intact sperm heads and tails (*see* Zhang et al., 2021 for images of this separation), as this fractionation process features sonication steps, the membrane integrity of the isolated sperm heads was systematically assessed prior to their use for proteomics. This analysis was performed using peanut agglutinin lectin (PNA) and acrosin antibodies to investigate acrosomal status. PNA is a well characterized marker of the outer acrosomal membrane (Flesch et al., 1998) and acrosin localizes to the acrosome matrix and inner acrosome membrane of boar sperm, respectively (de Vries et al., 1985; Bozzola et al., 1991). Compared to whole spermatozoa (non-sonicated sperm cells; WS), there was a visible loss of PNA signal in the sonicated sperm heads, as well as a loss or disruption to the acrosin localization (Figures 2A,B). This indicated that there is likely to be a disruption to the outer acrosomal membrane and potentially to the acrosomal matrix due to the head isolation protocol. In summary, these data indicated some exterior membrane damage to the sperm heads and tails following the separation procedure. For this reason, the proteomic characterization of the head and tail fractions in this manuscript should be used primarily for comparative purposes to differentiate the proteins that are enriched in the PT structure. This is unlikely to be a physiological inventory of all proteins contained within the boar sperm head and tail as membrane protein detection may have been impeded by the requisite cell preparation method.

### Proteomic Characterization of Boar Sperm Subcellular Fractions

**FIGURE 3:**
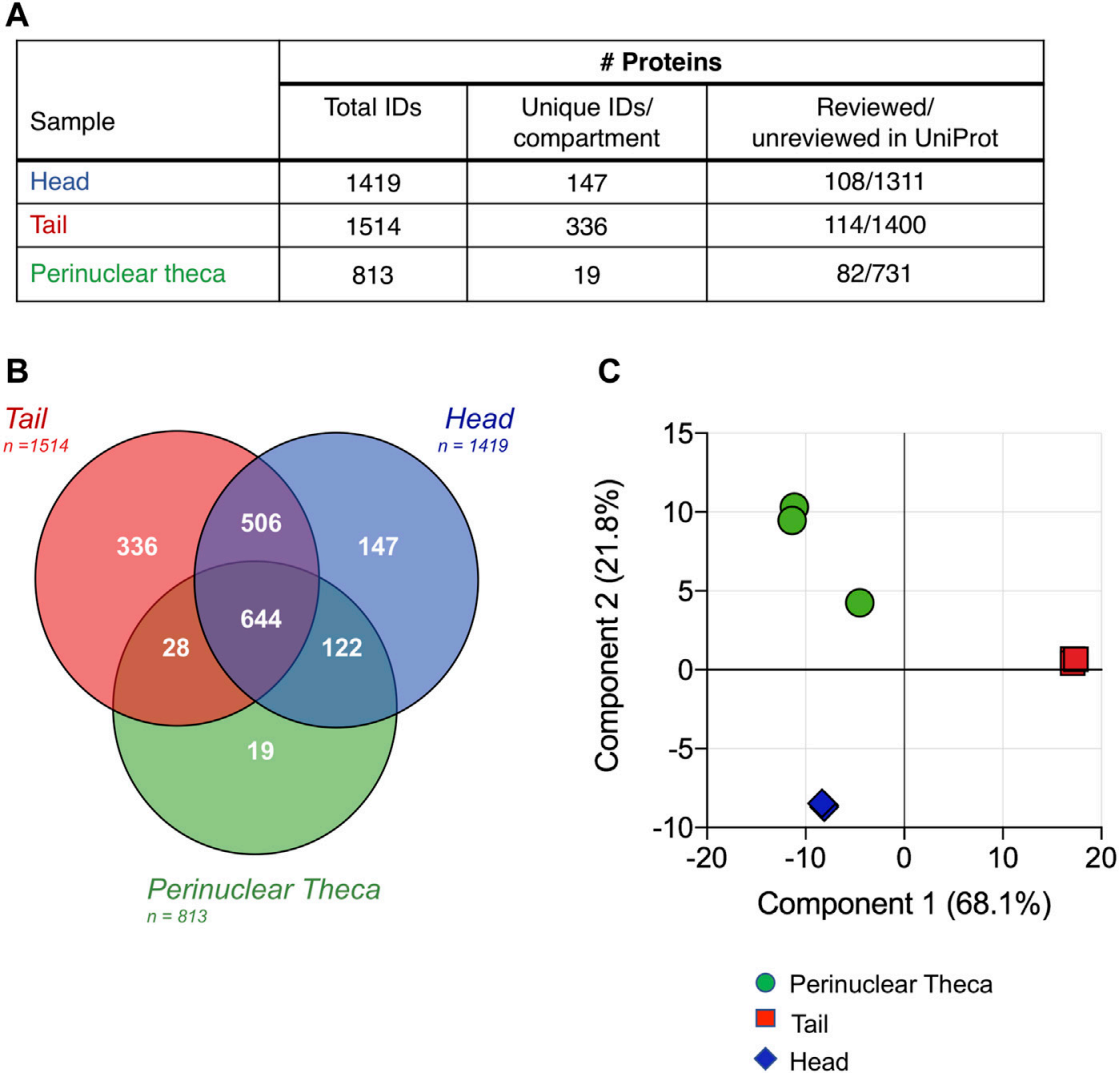
Characterization and comparison of boar sperm head, tail and PT protein compositions. **(A)** Following the restriction of each proteomic list based on the presence of each protein in all three replicates and strict quality control cutoffs, analysis of the head, tail and PT proteomes revealed 1,419, 1,514, and 813 proteins, respectively. Due to the limited annotation of the *Sus scrofa* proteome, a majority of the proteins featured in each sperm compartment held an “unreviewed” status within the UniProt database. **(B)** Proteins detected in each cell compartment were compared using a Venn diagram and principal component analysis **(C)** revealing the close clustering of biological replicates.

Proteomic characterization of the isolated head and tail components of boar spermatozoa revealed a total of 1,419, and 1,514 proteins identified in sperm heads and tails, respectively (Figure 3A). Notwithstanding any disruption to membrane proteins due to the cell separation method, these data represent an unparalleled depth of coverage for the boar sperm proteome with an identification of 1,802 proteins in total (Supplementary File S1). Notably, the entire proteome of *Sus scrofa* currently consists of just 1,438 reviewed proteins. Within the minute structure of the PT itself we identified 813 proteins of which only 10% feature in the reference proteome for *Sus scrofa* and carry a reviewed protein status in UniProt (Figure 3A). This implies that many of the proteins contained within this cellular compartment may be cell-type specific and are not well characterized. Intriguingly, our analysis indicated that the PT structure encompasses 57% of all the proteins detected within the whole boar sperm head. This reveals a new understanding of the extent of protein compartmentalization that takes place within spermatozoa during the final phases of spermiogenesis in elongating spermatids when the PT structure is formed (Longo and Cook, 1991).

Despite the highly compartmentalized morphology of the sperm cell, our analysis revealed that a total of 1,150 proteins were shared between the head and tail, accounting for 81% of head proteins and 76% of tail proteins (Figure 3B). Thus, a total of 147 proteins were identified that were unique to the head and 336 proteins that were unique in the tail (Figure 3B and Supplementary File S1). While we expected that all PT proteins would also be accounted for in the sperm head fraction (as this fraction will contain the PT *in situ*), 47 proteins were identified in the PT that were not found to be present in all three replicates of the head samples. This may be due to the isolation protocol for the PT facilitating the identification of less abundant proteins that are often difficult to detect in whole sperm or in sperm heads. This phenomenon has been described previously during a subcellular proteomic study of isolated human sperm nuclei where more than half of the identified proteins had not been detected in any previous whole sperm cell proteomic analysis (de Mateo et al., 2011).

To assess the reproducibility of the replicates and the extent of differentiation between the subcellular compartments, a principal component analysis (PCA) was conducted (Figure 3C). Although one PT replicate was found to vary marginally from the other two, the PCA confirmed the high degree of similarity between the three biological replicates analyzed for each sample group. This analysis also demonstrated the clear disparity between the proteomic constituents of the head, tail and PT compartments. Fittingly, samples from the head and PT were more closely associated than those of the head and tail or PT and tail, with component one accounting for 68.1% of the total variance between the samples.

### Composition of the Perinuclear Theca Proteome and Validation

**FIGURE 4:**
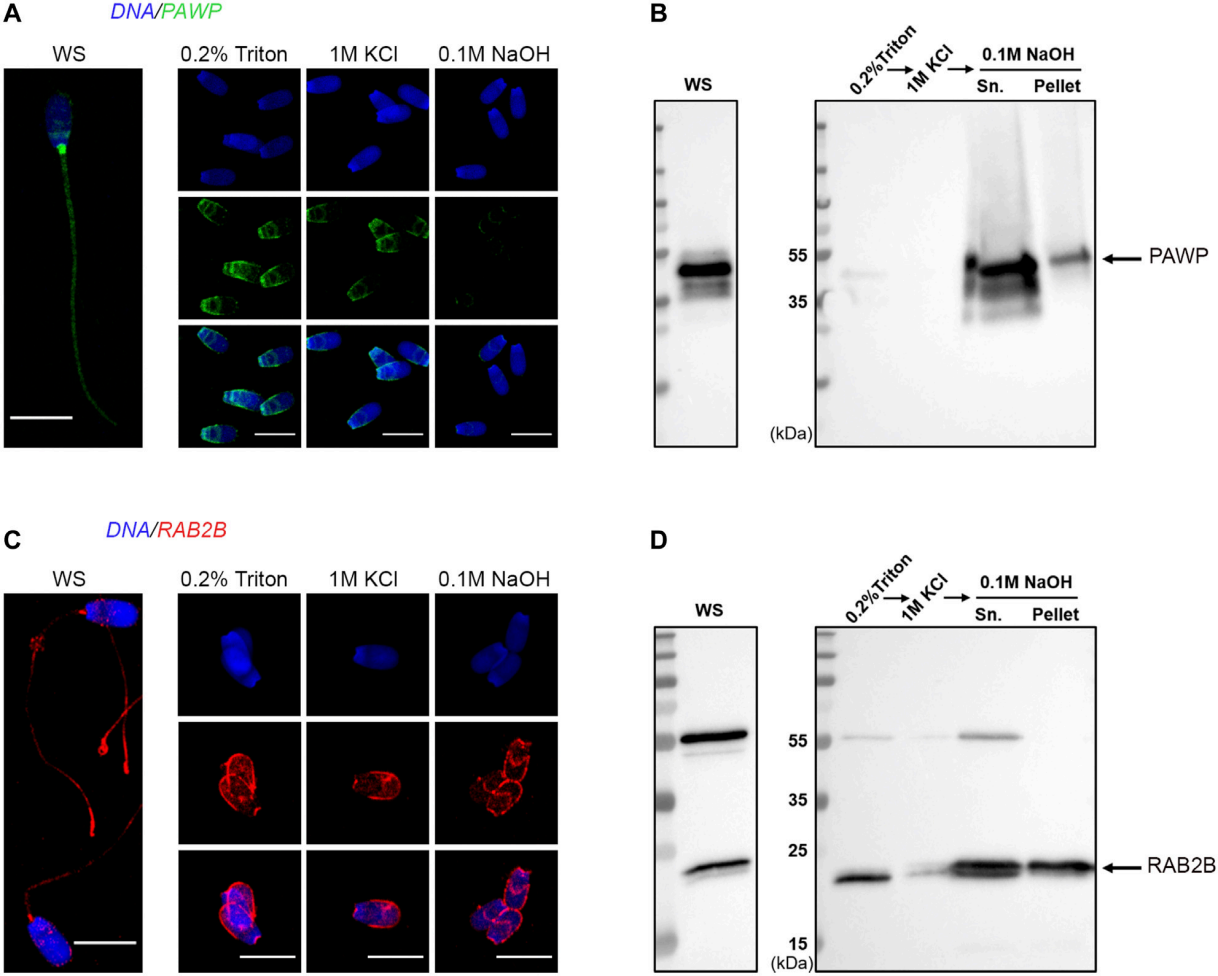
Validation of the presence of PAWP and RAB2B in the boar sperm PT. WS and sperm heads captured from each stage of the PT extraction protocol were examined through immunocytochemistry with antibodies to PAWP **(A)** and RAB2B **(C)**. Scale bar = 10 μm. Immunoblotting analysis of PAWP and RAB2B after the successive extractions and the resulting pellets (pellet) probed with anti-PAWP **(B)** and anti-RAB2B **(D)** antibodies.

**FIGURE 5:**
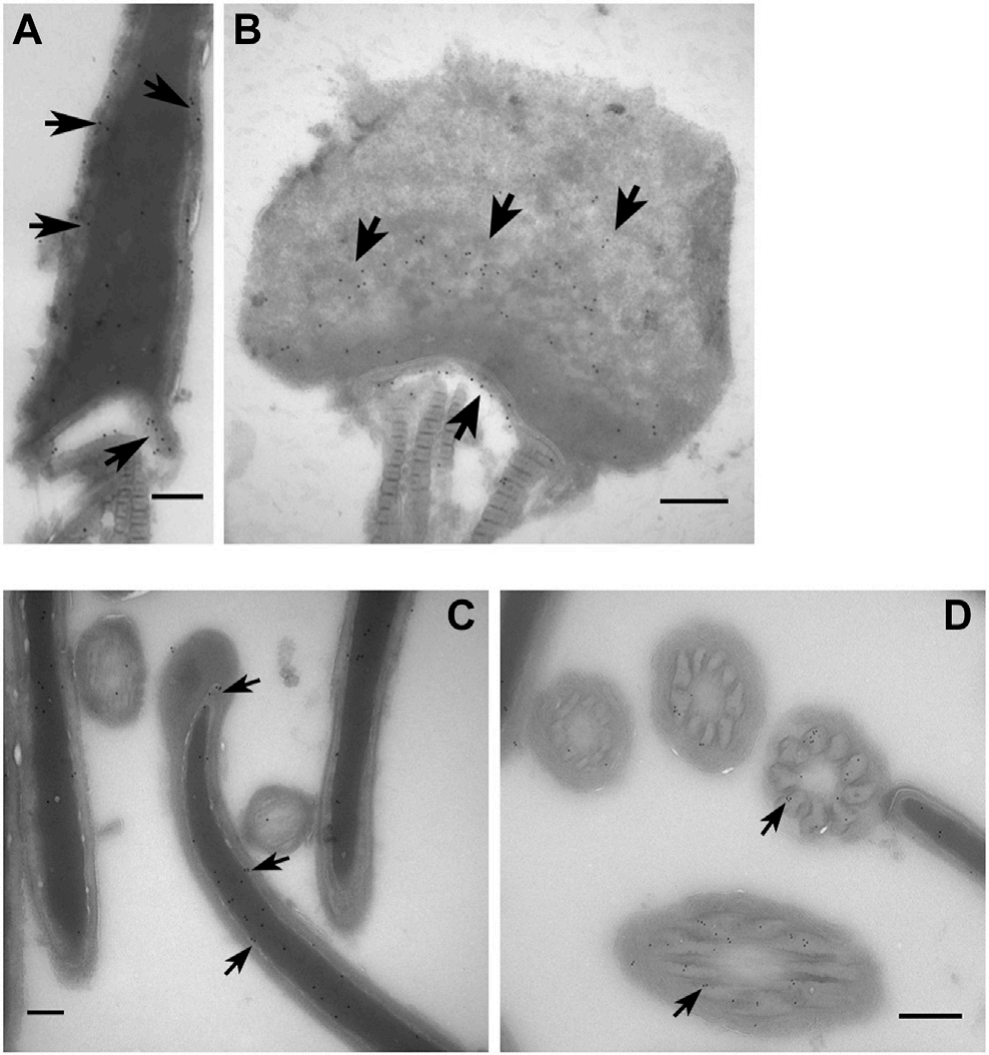
Ultrastructural localization of PAWP and RAB2B in boar spermatozoa. Immunogold labelling of PAWP on sagittal sections **(A)** and oblique sections through the post acrosomal sheath (PAS) region and **(B)** through a whole sperm head showing that PAWP localized to the PAS-PT and the connecting piece (arrow). **(C)** Immunogold labelling of RAB2B on sagittal section through a whole sperm head showing RAB2B localized to the sub-acrosomal layer and the sperm nucleus. **(D)** Immunogold labelling of RAB2B on cross sections of the sperm tail showing RAB2B localized to the outer dense fibers. Scale bar = 200 nm.

Within our PT proteome we identified well-known PT proteins that have been previously used to characterize this structure in other laboratories. These included: glutathione-S-transferase omega 2 (GSTO2); post-acrosomal sheath WW domain-binding protein (PAWP); several somatic histones including H2A, H2B, H3 and H4; as well as Ras-related protein Rab-2A and Ras-related protein Rab-2B (RAB2A; RAB2B). To further validate these data, we performed immunolocalization, immunoblotting and immuno-gold labelling experiments to confirm the presence of PAWP and RAB2B within the PT of boar spermatozoa. Immunolabelling of PAWP in whole sperm cells revealed a bright signal in the connecting piece with additional labelling in the post acrosomal region and sperm tail (Figure 4A). Immunolabelling in sperm heads after successive treatments with 0.2% Triton X-100, 1 M KCl and 0.1 M NaOH (as described in Figure 1) revealed that PAWP immunofluorescence was retained in the post acrosomal region and the equatorial segment of sperm heads after 0.2% Triton X-100 and 1 M KCl extraction. However, barely any PAWP signal was observed after 0.1 M NaOH treatment (Figure 4A). The additional foci of PAWP labeling on the equatorial segment of the sperm heads that was not present in the whole sperm cell staining is likely due to 0.2% Triton X-100 treatment before fixation exposing the epitope of this antigen. Immunoblotting analysis of PAWP under the same conditions revealed that PAWP was resistant to 0.2% Triton X-100 and 1 M KCl extraction, however, the majority of PAWP was recovered in 0.1 M NaOH extraction. A small residual amount of the protein was not solubilized and associated with the pellet; representing the sperm nuclear fraction (Figure 4B). Immunogold labeling of PAWP on ultrathin sections confirmed that PAWP was localized to the perinuclear theca between the plasma membrane and the nuclear envelope as well as in the connecting piece (Figures 5A,B). Taken together, these data confirm that PAWP is a specific PT residing protein in the boar. Immunostaining of RAB2B in whole sperm cells revealed a bright signal in the connecting piece and the tail of the sperm, but a weak signal in the sperm head (Figure 4C). However, immunostaining in sperm heads after successive treatments with 0.2% Triton X-100, 1 M KCl and 0.1 M NaOH revealed a stable and bright signal for RAB2B surrounding the sperm nucleus (Figure 4C). Additionally, immunoblotting analysis was used to evaluate the solubility of RAB2B from the sperm head. It was observed that besides the expected ∼23 kDa protein, an additional ∼55 kDa band was also detected in the whole sperm cells (Figure 4D). Immunoblotting for the successive treatments revealed that RAB2B was partially solubilized by 0.2% Triton X-100 and very small amount was also detected through 1 M KCl extraction (Figure 4D). The 0.1 M NaOH treatment extracted a moderate amount of RAB2B with some still associated with the sperm nucleus (Figure 4D; pellet). It was observed that the relative ratio between the ∼55 kDa and the ∼23 kDa bands was altered between whole sperm cells to sperm heads indicating that the ∼55 kDa was likely associated with the sperm tail (Figure 4D). This is fitting with the immunostaining of RAB2B in the whole sperm cells where strong RAB2B labeling was observed in the sperm tail (Figure 4C). Immunogold labelling of RAB2B on ultrathin cryo-coupes revealed that RAB2B was localized to and between the inner acrosomal membrane and the nuclear envelope indicating that RAB2B was residing in the sub-acrosomal layer of the perinuclear theca (Figure 5C). RAB2B also showed intensive labelling in the sperm nucleus as well as in the outer dense fibers (Figures 5C,D).

**FIGURE 6:**
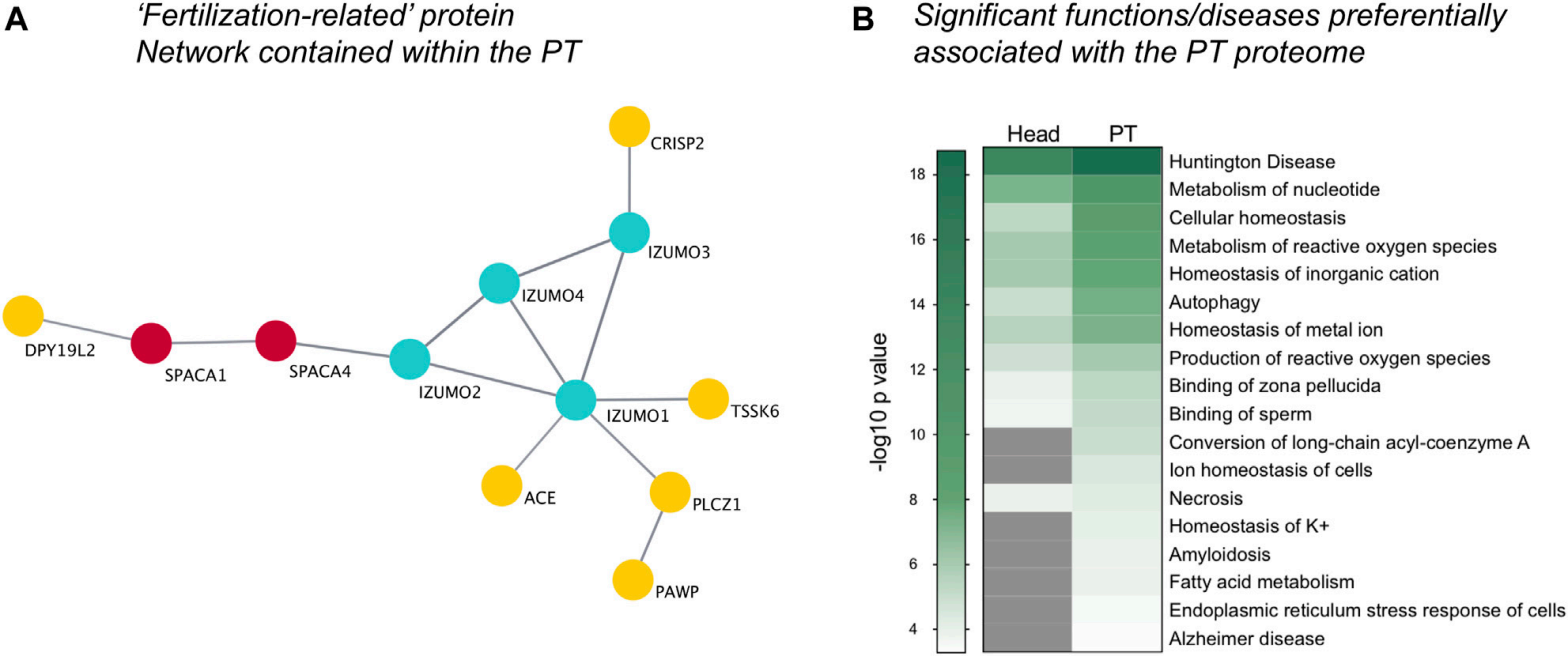
Visualization of fertilization-related proteins and functional pathway analysis. Within the whole PT proteome, a distinct protein cluster comprising key proteins involved in fertilization was detected through String protein network analysis and visualized using Cytoscape **(A)**. **(B)** Ingenuity Pathway Analysis software revealed the key diseases and functions enriched within the PT compared to the sperm head (−log10 *p*-value ≥ 1.3; *p*-value ≤ 0.05), featuring diseases with a basis in protein misfolding and functions related to the endoplasmic reticulum stress response, cellular homeostasis and fertilization, including “Binding of sperm” and “Binding of zona pellucida.” Grey indicates no significant value.

In addition to the previously characterized PT proteins validated above, our analysis revealed an extensive inventory of proteins known to be important for successful sperm-egg recognition and fertilization that reside within the PT structure (Supplementary File S1). Moreover, network analysis revealed that a subset of these fertilization-related proteins have the potential to interact within the PT (Figure 6A). This putative protein network included: Izumo sperm-egg fusion proteins 1–4 (IZUMO1-4), PLCZ1, cysteine-rich secretory protein 2 (CRISP2), testis-specific serine/threonine-protein kinase 6 (TSSK6) and several other related proteins (*see* Figure 6A). The detection of these fertilization-related proteins within the PT is congruous with the previously identified role for the PT structure in the process of sperm-egg interaction. Notably, we have recently confirmed that CRISP2 is indeed present within the PT of boar spermatozoa and may play a role in protein scaffolding/protein complex formation at this site (Zhang et al., 2021).

Additional characterization of the PT proteome using Ingenuity Pathway Analysis (IPA) software (Qiagen), revealed an increased enrichment for key fertilization-related functions in the PT compared to the sperm head proteome. These included the functions: “binding of zona pellucida” and “binding of sperm” (Figure 6B in which a −log10 *p*-value ≥ 1.3 indicates significance). Intriguingly, the PT proteome also supported several functions related to cellular homeostasis including “metabolism and production of reactive oxygen species,” “autophagy,” “ion and potassium homeostasis,” “fatty acid metabolism,” “amyloidosis” and “endoplasmic reticulum (ER) stress response in cells.” Moreover, several diseases linked to protein folding and ER stress were also found to be preferentially related to the PT proteome compared to that of the head. These diseases include “Huntington disease” and “Alzheimer disease” (Figure 6B) as well as related diseases such as chorea and cataract formation (data not shown) that are all underpinned by protein aggregation/misfolding and cellular stress. In keeping with this ER-like functional enrichment within the PT proteome, the well characterized ER-resident proteins calreticulin (CALR), calnexin (CANX), protein disulfide-isomerase A3 (PDIA3/ERp57) were all found to be present in the PT. For a full list of proteins detected within the isolated PT *see* Supplementary File S1.

### Significantly Enriched Proteins and Pathways in the Perinuclear Theca Compared to the Head and Tail

**FIGURE 7:**
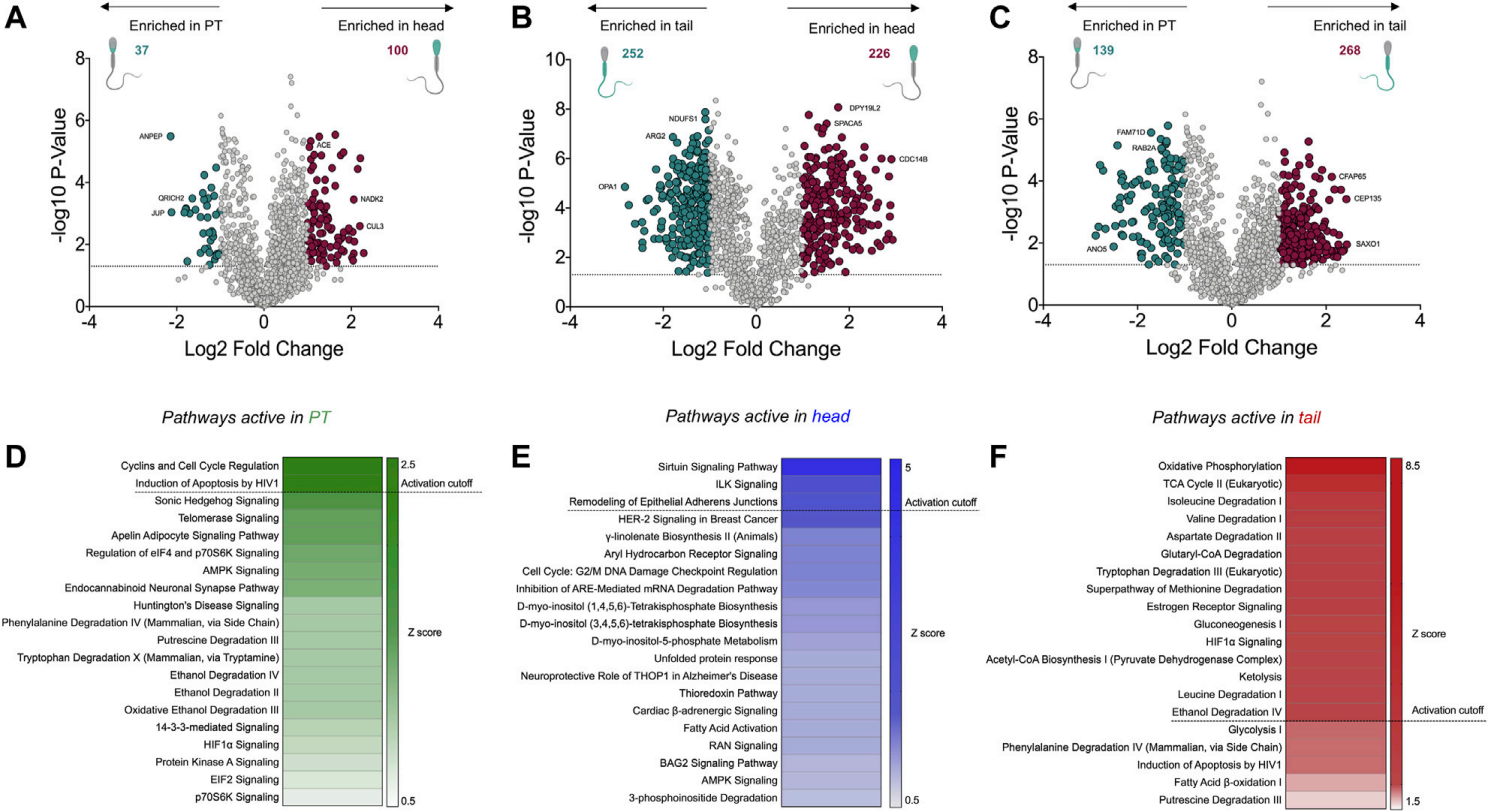
Significantly enriched proteins within the perinuclear theca compared to the sperm head and tail, and functional analyses of the cell compartment proteomes. **(A** **–C)** Fold changes and *p*-values were calculated from abundance values for each protein detected in the perinuclear theca (PT), head and tail. In this analysis a protein was deemed to be significantly enriched in each sample type upon meeting a fold change cut off of ≥2 and a *p*-value of ≤0.05 (dotted line). In the volcano plot visualizations these cut offs are equivalent to a log2 fold change of ≥1 and a −log10 *p*-value ≥1.3. Proteins that meet these criteria were designated colors (red and green) in panels **(A** **–C)**. **(D** **–F)** The Z-score function within Ingenuity Pathway Analysis (IPA) software was used to predict active pathways within each sample type. A Z-score ≥2 indicates the likely activity of the pathways listed in panels **(D** **–F)**.

While the analyses conducted above yielded intriguing insight into the functions of the PT proteome, many of the proteins contained within the PT were also found to be present within the sperm head proteome and may play roles in both compartments. Thus, to specifically explore the enriched proteins within the PT compared to the sperm head and tail fractions, statistical analyses were employed to calculate fold changes and *p*-values between each permutation. This analysis provided statistical confidence to aid in determining which proteins are most likely to partition into the PT compared to the head or tail, and thus may play crucial roles in the formation and/or function of this structure. Represented by volcano plots, this analysis revealed 37 proteins that were significantly enriched with a fold change ≥ 2 in the PT compared to the head (Figure 7A and Supplementary Table S2). Of the characterized proteins within this list, those with the most substantial fold changes included aminopeptidase (ANPEP), and glutamine rich 2 (QRICH2), the latter of which has been shown to play an important role in sperm cell structural development and fertility (Shen et al., 2019). When comparing the PT to the tail, 139 proteins met our significance cut offs indicative of enrichment within the PT compared to the tail (Figure 7C and Supplementary Table S3). As expected, the most substantial compartmentalization was demonstrated by comparing proteins enriched in the head compared to the tail (Figure 7B) where several hundred proteins made the significance cut offs we applied, signifying the distinct proteomic stratification which takes place in these structurally and functionally diverse cellular compartments.

To improve our understanding of the function of the PT within sperm cells, differentially expressed boar sperm proteins were mapped to their human homologs (Supplementary File S1) and pathway analysis was performed using IPA. For this analysis we utilized the Z-score algorithm within IPA to indicate pathways that are likely to be more active in the PT compared to the sperm head or tail (a Z-score cut off ≥ 2 indicates likely active pathways; Z-scores between 1.5 and 2 indicate a trend towards active). The most significant canonical pathway active in the PT compared to the sperm head was related to “cyclins and cell cycle regulation” (Figure 7D), which is a key pathway associated with spermiogenesis (Wolgemuth and Roberts, 2010). Several members of this pathway were detected in the PT including: serine/threonine-protein kinase ATR (ATR), glycogen synthase kinase-3 beta (GSK3B) and histone deacetylase 11 (HDAC11). Each of these proteins are also known to play roles in cell death pathways such as apoptosis. Notably, pathway analysis also revealed that apoptosis-associated pathways such as those that occur in response to HIV1, are likely to be active (Figure 7D). Comparison of the head and tail proteomes using IPA revealed the putative activity of several well-characterized signaling pathways including sirtuin signaling and ILK signaling (Figure 7E). Conversely, the tail fractions were enriched in pathways relating to cell metabolism and mitochondrial dynamics including oxidative phosphorylation, the TCA cycle and amino acid degradation (Figure 7F). These are the key pathways involved in ATP production within the mitochondria of mammalian sperm cells (Piomboni et al., 2012).

### Perinuclear Theca Protein Network Enrichment Analysis

**FIGURE 8:**
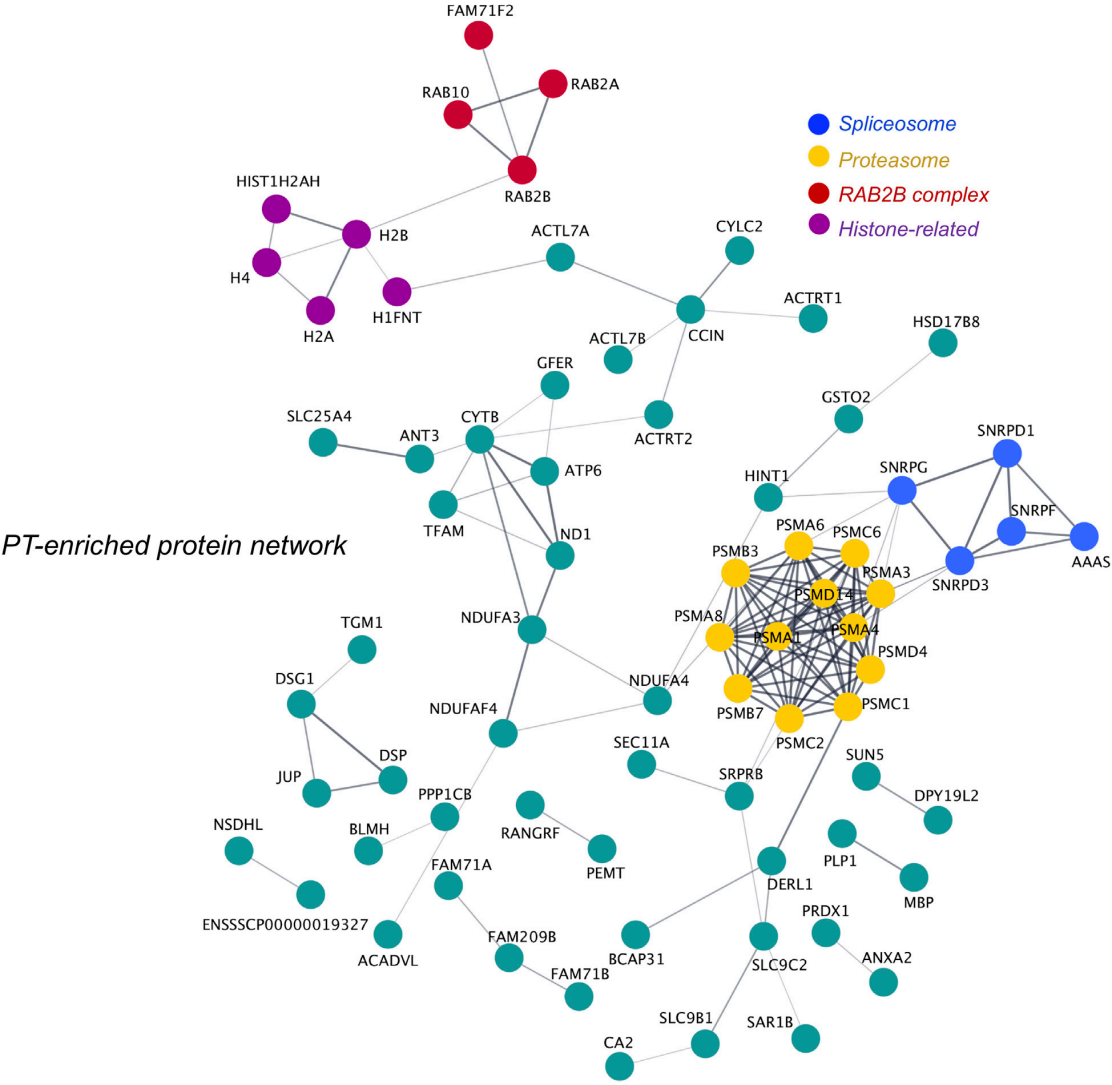
Protein network analysis of the perinuclear theca and identification of key protein clusters. All proteins deemed to be enriched in the perinuclear theca (PT) compared to the sperm head and tail were searched in the online protein interaction database String *via* their *Sus scrofa* UniProt protein accessions alongside the 19 proteins that were found to be unique to the PT compared to the head and tail. The protein network formed by the PT proteins was then exported to Cytoscape and protein clusters were manually designated functional annotations based on literature searches conducted on their protein components. This analysis revealed four proteins clusters enriched in proteins related to the “proteasome” (yellow) and “spliceosome” (blue), somatic histones (histone-related; purple), RAB proteins (RAB2B complex; red). Single proteins that did not interact with other PT proteins within the network feature in Supplementary Figure S1A.

As the PT is a highly dense structure known to feature extensive protein scaffolding and support high molecular weight protein complex formation (Hess et al., 1995; Lécuyer et al., 2000; Zhang et al., 2021), we investigated putative protein-protein interactions within the boar sperm PT proteome. Specifically, using the tools String and Cytoscape we constructed a network featuring all PT proteins that were either significantly enriched (*p* ≤ 0.05; fold change ≥ 2), or unique to the PT structure compared to the head and tail. This network analysis revealed the distinct clustering of numerous proteins within the PT (Figure 8) with a majority of proteins putatively linked to at least one partner protein within the network (for singletons that did not form putative interactions *see* Supplementary Figure S1A). As expected, two distinct clusters within the PT network were formed by the RAB proteins and somatic histone proteins (referred to as “RAB2B complex” and “histone-related”). However, additional enriched protein clusters were revealed through this analysis with several members of the proteasome forming a large, distinct cluster within the PT network, and several spliceosome-related proteins forming a small cluster (Figure 8). In regard to the proteasome-related cluster, the specific presence of PSMA8 suggests that this protein cluster best resembles the spermatoproteasome, a structural variant of the proteasome that is formed to promote the degradation of acetylated histones during spermiogenesis (Qian et al., 2013).

**FIGURE 9:**
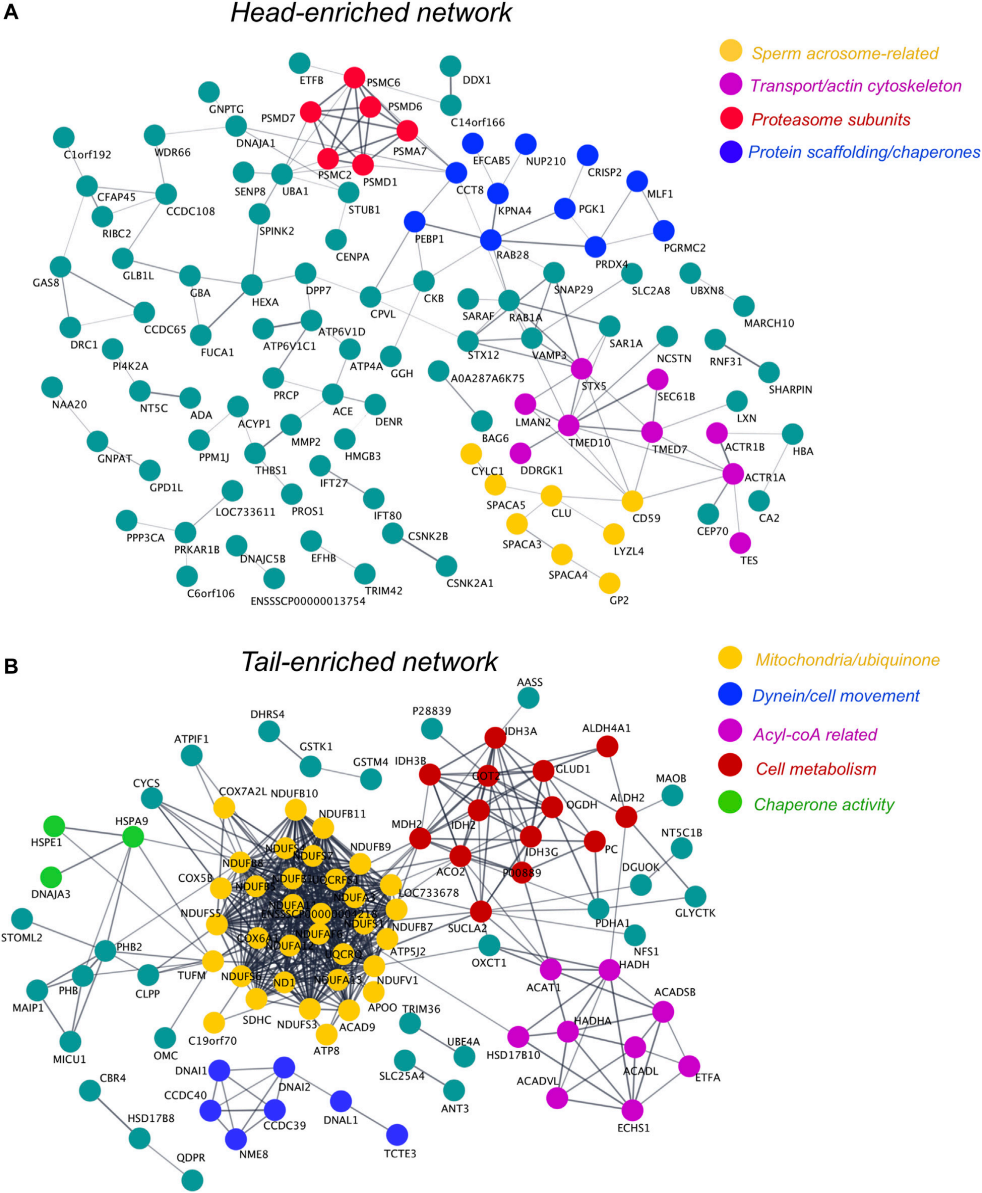
Protein network analysis of head- and tail-enriched proteins within boar spermatozoa and identification of key protein clusters. To form each protein network, proteins significantly enriched in the sperm head **(A)** or tail **(B)** compared to the PT were uploaded *via* their *Sus scrofa* UniProt accessions to the online protein interaction database String. These protein networks were then exported to Cytoscape and protein clusters were manually designated functional annotations through literature searches of their protein components. Due to the limited annotation of the *Sus scrofa* proteome, several uncharacterized proteins feature in these networks. In the absence of an assigned gene name, these uncharacterized proteins were labeled with their UniProt accession number where possible. Single proteins that did not interact with other proteins within the head or tail networks can be found in Supplementary Figures S1B,C.

To delineate the role of proteins that were significantly enriched in the sperm head and tail compared to the PT, network analysis was also performed for these cell compartments. For the sperm head, network analysis revealed the presence of a cluster of proteins involved in formation and function of the sperm acrosome, protein transport and the actin cytoskeleton (Figure 9A). Complementing our analysis of the PT, the sperm head also featured some additional proteasome subunits to those detected in the PT, as well as a distinct cluster of proteins with molecular chaperone and protein scaffolding activity. In the sperm tail network, we observed extensive protein clustering of key components involved in the sperm mitochondria/ubiquinone and in cell metabolism and acyl-coA (Figure 9B). In addition to this, the tail network was also enriched in proteins related to cell movement/dyneins, as well as chaperone activity (Figure 9B). For access to the singleton proteins that were enriched in each cell compartment but not featured in the networks *see* Supplementary Figures S1A–C.

## Discussion

The perinuclear theca (PT) is a unique structure within the sperm head that has been reported to house proteins critical for sperm-egg interaction and provide structural support to the nucleus during the long process of sperm transit through the reproductive tracts (Sutovsky et al., 2003; Oko and Sutovsky, 2009). Notwithstanding several highly valuable studies of the PT and subsequent in-depth characterization of several PT-associated proteins including; post-acrosomal sheath WW domain-binding protein (PAWP) and glutathione S-transferase omega 2 (GSTO2) (Wu et al., 2007; Hamilton et al., 2017), the full proteomic content of the PT relative to other sperm components has not been explored using contemporary mass spectrometry approaches. In the current study we have taken advantage of the latest updates to the *Sus scrofa* proteome and sperm subcellular fractionation strategies established previously (Oko and Sutovsky, 2009) to conduct a comprehensive characterization of the PT proteome. Our study identified and quantified 1,419 proteins in the sperm head, 1,514 proteins in the sperm tail and 813 proteins in the PT providing a valuable resource for the field of reproductive science. Moreover, our analysis revealed that the majority of proteins recently confirmed to play essential roles in sperm-egg fusion are indeed housed within the PT in the mature sperm cell. Herein we consider the individual proteins and protein networks identified in our characterization of the PT and discuss their relationship to known and less-well-known functions of the PT during mammalian fertilization.

The PT was isolated from the sperm head using a combination of detergents (0.2% Triton X-100, 1 M KCl and 100 mM NaOH) previously established to extract PT proteins from bull (Aul and Oko, 2002), rat (Oko, 1995), mouse (Korley et al., 1997), human and boar spermatozoa (Lécuyer et al., 2000). Within this protocol, the final alkaline extraction step is thought to extract all the covalently bound PT proteins. Similarly, some proteins that interact with polar bonds to the PT, such as histones, are detached during the saline extraction step (Tovich and Oko, 2003; Hamilton et al., 2021). Somatic histones have been identified as major constituents of the PT of bovine (Tovich and Oko, 2003) and murid spermatozoa (Hamilton et al., 2021), and those somatic histones are extractable using 1 M KCl. However, the extractability of the histones differs in our study whereby somatic histones were still abundantly present in the final alkaline step after high salt treatments. It is unlikely that the histones detected are residual sperm chromatin bound histones as the sperm nucleus has been reported to remain condensed after alkaline treatments. Moreover, Hamilton et al., have recently demonstrated that the core somatic histones detected in the mouse sperm PT are *de novo* synthesized in round spermatids (Hamilton et al., 2021). These authors were also able to demonstrate a role for such histones post-fertilization using ICSI (Hamilton et al., 2021). While similar experiments would need to be performed in boar sperm to verify that our findings are congruous, the remarkable conservation of somatic histones residing in the PT in eutherian sperm cells points to an important role for the PT in housing these proteins prior to fertilization.

In addition to the identification of histone proteins within the PT, our proteomic analysis revealed the presence of numerous RAB family proteins within the PT, some of which were previously reported by Oko (Mountjoy et al., 2008). RAB proteins have more than 60 family members in the human genome making it the largest branch of the Ras-related family of small-GTPases (Bock et al., 2001). There are several well-established functions of RAB proteins including; participation in vesicle trafficking pathways such as membrane tethering; vesicle budding and regulation of vesicle movement; along with cytoskeletal functions (Zerial and McBride, 2001). Most RAB proteins have universal expression, with few RAB proteins having biased expression specific to cells or tissues. Related to reproduction, 25 RAB proteins have been reported to play key roles in female meiosis and 12 RAB proteins function in male meiosis (Shan and Sun, 2021). In the present study we have identified seven RAB proteins (RAB2A, RAB2B, RAB10, RAB14, RAB17, RAB18 and RAB39A) in the PT. Of these proteins, RAB2A and RAB2B are the most well characterized. A previous report in bull spermatozoa has indicated that RAB2A is associated with the membrane of growing proacrosomic and acrosomic secretory vesicles during acrosomal biogenesis. RAB2A subsequently becomes a prominent subacrosomal layer (SAL)-PT protein in mature bull spermatozoa. The authors postulated that RAB2A-mediated vesicular pathways occurring during spermiogenesis may have little in common with those of somatic cells (Mountjoy et al., 2008). Given the high sequence similarity (93% based on UniProt alignment) between bovine RAB2A (A0A3Q1MHX8) and porcine RAB2A (A0A480X841) and the fact that RAB2A and RAB2B share 83% identity, it is very likely that RAB2B follows a similar developmental route of deposition during SAL-PT assembly in developing boar spermatozoa. Notably, our data also revealed that RAB2B, RAB10 and RAB17 were enriched in the PT and that these proteins can potentially form a distinct protein cluster or putative complex within the PT (Figure 8). RAB10 is known to colocalize with its regulator RAB GTPase-activating protein in male germ cells within the manchette structure involved in spermatid head formation (Lin et al., 2017). While, germ cell roles are yet to be evaluated for RAB17, this protein is involved in basolateral to apical transcytosis (Striz and Tuma, 2016; Striz et al., 2018), melanosome exocytosis (Beaumont et al., 2011) and efferocytosis through the recycling endosome (Yin et al., 2016; Yin et al., 2019). A recent study has demonstrated that homotypic and heterotypic RAB-RAB protein interactions induce vesicle tethering on two opposing membranes (Segawa et al., 2019), adding strong evidence that RAB proteins can function diversely in membrane tethering. Given these data, it is possible that Golgi-associated RAB2 may be involved in anchoring of the acrosome to the nuclear envelope during sperm head formation through interaction with other RAB proteins and may thus serve as a protein scaffold to tighten the association of the acrosome with the nucleus. Certainly, the positioning of the RAB2 proteins within the PT is indicative that this hypothesis should be explored further in developing germ cells of the boar. Moreover, further experiments should be focused towards understanding the specific mechanisms of vesicle transport mediated by RAB proteins during spermiogenesis.

Within the PT, the ES is commonly considered the first site on the sperm cell that makes contact with the oolemma, subsequently exposing the PAS-PT to the oocyte cytoplasm (Sutovsky et al., 2003; Vjugina and Evans, 2008). In our study, we have uncovered an extensive protein network related to sperm-egg fusion within the PT (Figure 6A). Numerous studies have demonstrated that IZUMO1 is required for sperm-egg fusion (Inoue et al., 2005; Satouh et al., 2012; Bianchi et al., 2014). It is believed that the appearance of IZUMO1 in the post-acrosomal region of sperm head after the acrosome reaction is indispensable for sperm-egg fusion. Consistent with the network we have described here, testis-specific serine kinase (TSSK6) has been reported to be involved in the re-localization of IZUMO1 (Sosnik et al., 2009). Intriguingly, two of the best-described potential sperm oocyte-activating factor candidates, phosphoinositide specific phospholipase ζ (Saunders et al., 2002) and PAWP (Aarabi et al., 2014), were also present in this PT protein network related to fertilization. In terms of validation, our data also revealed that PAWP was indeed localized to the PAS-PT as well the ES, and was alkaline extractable indicating its presence in PT. Additionally, DPY-19-like 2 (DPY19L2), a key causative factor related to human globozoospermia (Koscinski et al., 2011) was also found in the PT network. DPY19L2-deficient human spermatozoa have dramatically decreased protein expression, with the modulated proteins including: sperm acrosome associated 1 (SPACA1), IZUMO1, and PLCZ1 (Guo et al., 2019). Our findings provide evidence that these proteins are all likely to be present within the PT structure of spermatozoa and thus may be in close proximity to DPY19L2. Aberrations specific to the PT-proteome and PT structure in response to DPY19L2 deficiency will be interesting to explore. Finally, of the proteins within this fertilization-related network we have recently confirmed that cysteine rich secretory protein 2 (CRISP2) is indeed present in the PAS-PT of boar spermatozoa and is involved in protein complex formation and potential protein scaffolding at this site in which the free thiols on cysteine residues play an important role (Zhang et al., 2021). Taken together, the sperm-egg fusion related network we have documented within the PT is congruous with several previous studies described above and provides a novel basis to understand functional crosstalk between cytosolic molecules within the cytoskeleton.

Intriguingly, pathway analysis of the proteins abundant in the PT compared to the head and tail revealed a surprising enrichment of endoplasmic reticulum (ER) proteins residing in the PT. It is held that during testicular sperm maturation, spermatozoa lose most of their cytoplasmic content and many organelles including the ER. Although there are ER proteins added to the sperm surface during epididymal transit (Guo et al., 2007), it is unlikely that those ER proteins end up in the PT, since the PT is formed earlier during spermiogenesis. Supporting this, two ER resident proteins, calreticulin (CALR) and protein disulfide isomerase A3 (PDIA3/ERp57) are known to be present in the developing acrosome of haploid spermatids and are retained in the acrosomes of mature rat spermatozoa (Nakamura et al., 1993; Ohtani et al., 1993). ERp57 interacts with CALR and CANX, the ER chaperones, promoting glycoprotein folding in somatic cells (Ellgaard and Helenius, 2003) as well as testicular germ cells (Tokuhiro et al., 2012). Furthermore, ERp57 is associated with the ES of acrosome reacted sperm in rat (Liu et al., 2014), mice (Ellerman et al., 2006) and human (Zhang et al., 2007), indicating a role in sperm-egg fusion. Functioning as a multifunctional thiol-disulfide oxidoreductase, ERp57 has been speculated to trigger conformational changes in the proteins participating sperm-egg fusion (Ellerman et al., 2006; Zhang et al., 2007). Taken together, the PT enrichment of ERp57, CALR and CANX, amongst other ER proteins, suggests that these proteins may be stored in the PT to ensure their correct position to play a role in initiating the sperm-egg fusion process. This hypothesis requires explicit validation by tracking the localization of ER proteins throughout spermiogenesis and during the process of fertilization.

The PAS-PT is the first sperm head structure that is degraded within the ooplasm after fertilization (Sutovsky et al., 2003). Thus, it has been proposed by others that the PT may harbor sperm-borne molecules necessary for zygotic development or degradation processes. Previous investigations of the sperm borne factors present in the PAS-PT have demonstrated their intriguing roles in oocyte activation, pronuclear formation and early embryo development (Saunders et al., 2002; Wu et al., 2007; Aarabi et al., 2014; Hamilton et al., 2019). It is likely that, before exerting their functions during gamete fusion and early embryo development, these factors must first become soluble to be able to diffuse through the ooplasm. Thus, the rapid disassociation of PAS-PT after sperm-egg interaction may be indispensable to release the cytosolic molecules from the PT. Importantly, reduction of the disulfide bonds and the activity of glutathione are important for sperm head and nuclear decondensation (Perreault et al., 1984; Sutovsky and Schatten, 1997). GSTO2, an oxidative-reductive enzyme residing in the PAS-PT has been reported to play a role in removal of the PAS-PT structure, accelerating sperm nuclear decondensation (Hamilton et al., 2019). In line with this role, our data have demonstrated that CRISP2, localized to PAT-PT of the boar sperm head, is capable of forming disulfide bond-sensitive protein complexes at this site (Zhang et al., 2021). Moreover, our unpublished observations indicate a rapid dispersal of the PT after sperm entry in the oocyte i.e. before the sperm nucleus is decondensing and before mitochondria are degraded. This suggests that the reduction of the protein disulfide bridges may allow solubilization of the PT contents which then can diffuse through the oocyte’s cytosol and induce oocyte activation. The dispersal of the PT in the oocyte’s cytoplasm also allows the subsequent sperm nuclear chromatin decondensation in order to form the male pronucleus. Oxidation of protamine thiols is crucial for sperm chromatin condensation during sperm maturation and protecting the sperm heads against physical damage and DNA oxidation (Seligman et al., 1994; Pfeifer et al., 2001). A specific sperm nucleus glutathione peroxidase is shown to be responsible for this process (Pfeifer et al., 2001). In addition to these putative roles in the initiation of sperm-egg fusion, the PT also acts as a naturally rigid protein layer protecting the sperm nuclear material during the journey of the sperm through the male and female reproductive tracts before encountering the oolemma.

Consistent with the rapid degradation of the PT that has been observed post-fertilization, network analysis of PT proteins revealed an enrichment of proteasome subunits. The proteasome or “spermatoproteasome” is known to be critical for several steps of mammalian fertilization, including sperm capacitation, acrosomal exocytosis and sperm-zona pellucida penetration (Zimmerman and Sutovsky, 2009). Moreover, the ubiquitin proteasome pathway has been implicated in mammalian spermatogenesis and fertilization (Sutovsky, 2003; Yi et al., 2007). Importantly, it has been demonstrated that both the ubiquitin-proteasome system and autophagy are involved in the degradation of the sperm mitochondria after fertilization in pigs (Song et al., 2016). Given that the PAS-PT and the sperm mitochondrial sheath are assembled using the same machinery (Kierszenbaum, 2002; Oko and Sutovsky, 2009), a similar degradation mechanism, analogous to mitophagy, may also apply to the PAS-PT post-fertilization. Strong support for this proposal was garnered through IPA predicting that autophagy is greatly activated in the PT compared to the sperm tail. The knowledge that autophagosomes are present in matured porcine oocytes (Lee et al., 2014; Shen et al., 2018) also raises the possibility that the autophagosomes present in oocytes are recruited to the sperm head after sperm-egg fusion and trigger sperm PT dispersion. Thus, we speculate here that both autophagy and the ubiquitin-proteasome system may aid in PAS-PT degradation post-fertilization in the ooplasm.

In summary, the present study has characterized for the first time the whole PT proteome of boar spermatozoa and evaluated protein abundance relative to the head and tail compartments. This analysis simultaneously confirms decades of in-depth analysis of the individual proteins housed within the PT, while providing an abundance of new information regarding the complexity of the PT structure. Moreover, our analysis of these data has allowed the generation of putative protein networks that exist in the PT and can now be investigated in the context of sperm-egg fusion and sperm cell development. However, there are important limitations to this study and reasons for caution that should also be considered when interpreting the data. This is the first time that a high resolution proteomics approach has been applied following the PT protein extraction protocol described above. While this extraction protocol has been long established in the field, there are several steps which may yield contamination of proteins from other cell compartments or membranes into the perinuclear theca fraction that may not have been detected in the original studies without mass spectrometry. Namely, the presence of nuclear proteins or nuclear membrane proteins in the PT, while congruous with previous studies, should be interpreted with caution and independently validated. Additionally, the head and tail separation technique, while highly efficient in yielding >95% of head-tail separation, may still yield some contamination that can also be detected through mass spectrometry. An example of this is the presence of translocase of outer mitochondrial membrane 34 (TOMM34) in the sperm head, while it would be expected to sub-fractionate into the tail fraction. Despite this, TOMM34 has been described to be predominantly present in the cytosol of various cells and is also involved in chaperone systems not only for protein folding and import into the mitochondria but also in the cytosol (Chewawiwat et al., 1999; Durech et al., 2016; Muller et al., 2019). The fact that this protein is not fully associated to mitochondria may well explain its presence in the sperm head fraction. Comparatively, true marker proteins of the mitochondrial matrix (such as succinate dehydrogenase subunits, *see* Supplementary File S1) are virtually devoid in the sperm head and PT fractions when compared to the sperm tail fraction. Nonetheless, we suggest that the use of proteins of interest from our PT list should be complemented by orthogonal localization studies to visualize their presence within the PT.

In summary, notwithstanding the caveats outlined above, our data support the notion that during its formation, the condensing PT attracts several proteins that are now identified in this study for their eventual use during and post-fertilization. Furthermore, the PT forms a protective structural layer surrounding the nuclear envelope that may be reinforced through the scaffolding and crosslinking actions of its proteomic constituents such as CRISP2. We speculate that after the disassociation of the PT prior to sperm-egg fusion, the liberated sperm proteins may provide or supplement existing machinery for paternal chromatin decondensation and pronucleus formation. This model fits well with biochemical data provided by the Sutovsky and Oko labs, and others, over the past two decades and contributes to the field by providing the identity of long sought after PT-enriched proteins that may be essential for the efficacy of the sperm-egg interaction.

## Data Availability

The data presented in the study are deposited in the ProteomeXchange Consortium via the Proteomics Identification Database (PRIDE) partner repository with the dataset identifier PXD030020.
