# Supplementary figures and images for: High Resolution Proteomic Analysis of Subcellular Fractionated Boar Spermatozoa Provides Comprehensive Insights Into Perinuclear Theca-Residing Proteins
Source: Front Cell Dev Biol. 2022 Feb 18;10:836208. doi: 10.3389/fcell.2022.836208 (PMC8894813; doi:10.3389/fcell.2022.836208)

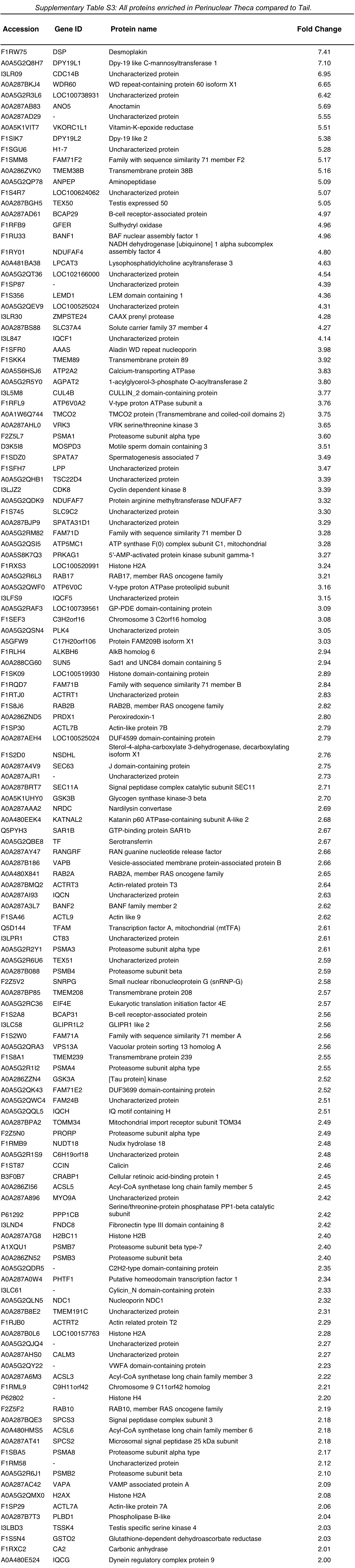

Supplement: Supplementary file 1 [file Image3.JPEG]

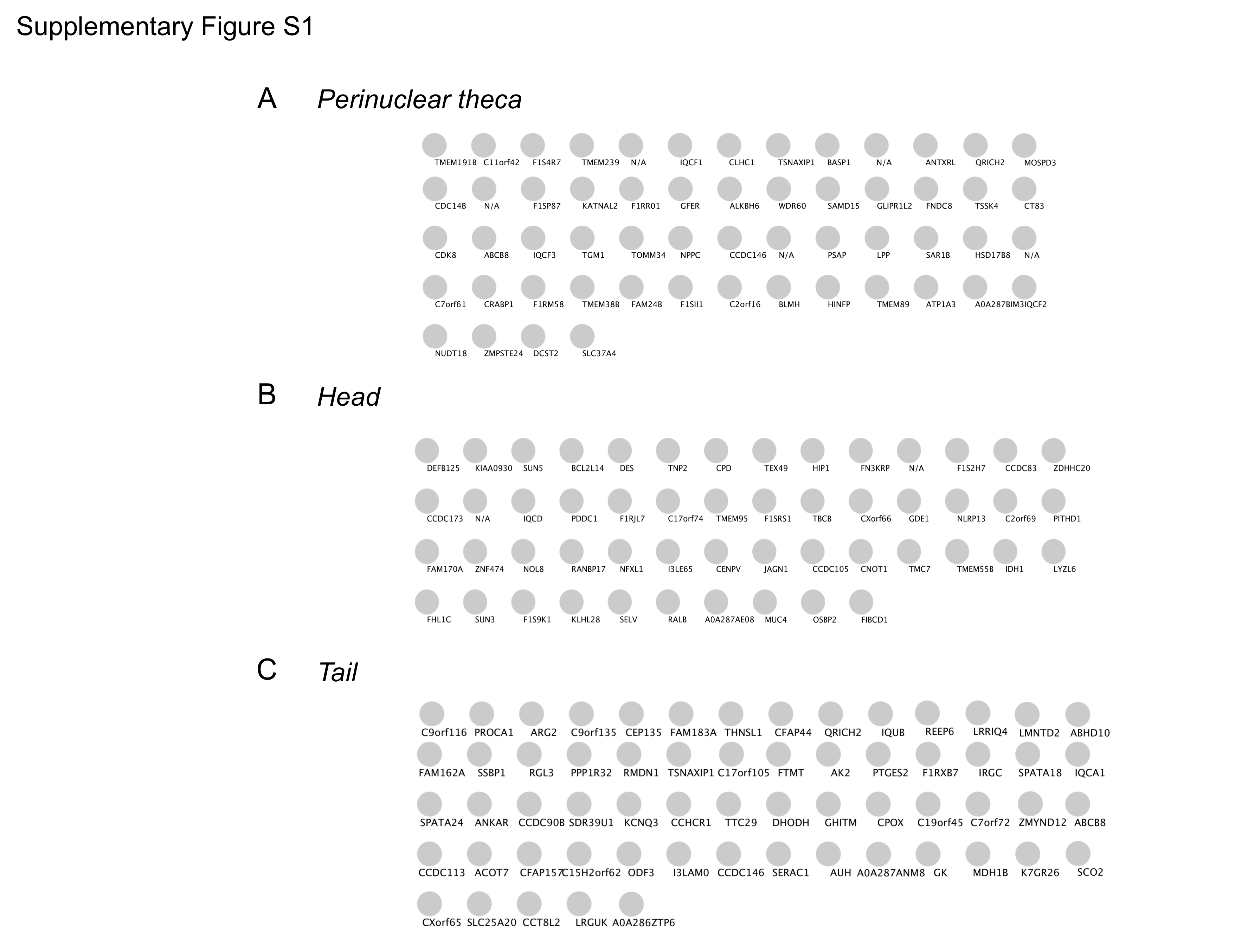

Supplement: Supplementary file 2 [file Figure10.JPEG]

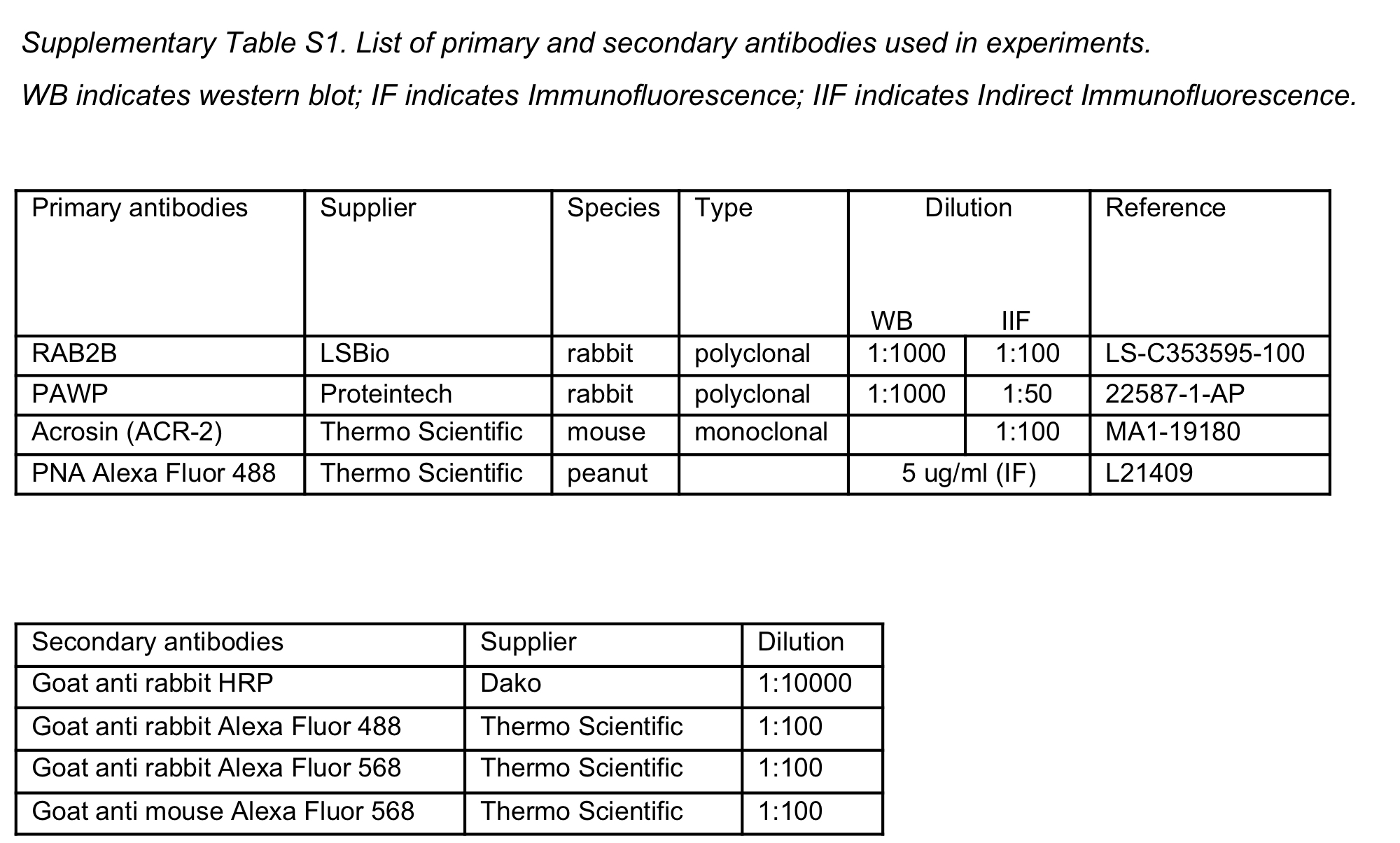

Supplement: Supplementary file 3 [file Image1.JPEG]

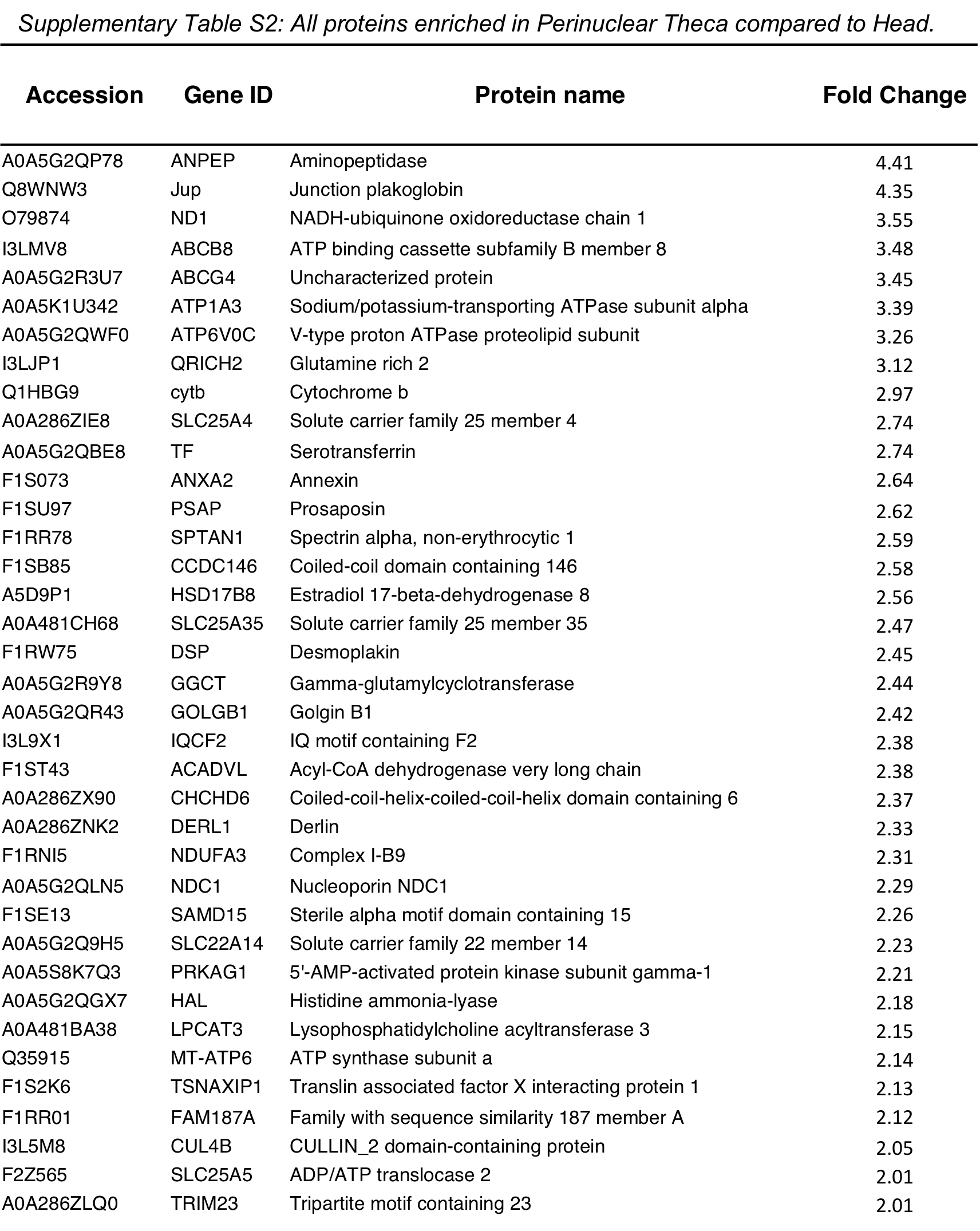

Supplement: Supplementary file 4 [file Image2.PNG]
